# Safety and efficacy of honeysuckle therapy for epidermal growth factor receptor inhibitor-related rash: a systematic review and meta-analysis

**DOI:** 10.3389/fphar.2026.1875649

**Published:** 2026-08-31

**Authors:** Li-Feng Liu, Shuang Liu, Jie Jiao, Fang-Yuan Zhang

**Affiliations:** 1 Department of Lung Neoplasms, Tianjin Medical University Cancer Institute and Hospital, National Clinical Research Center for Cancer, Tianjin, China; 2 Key Laboratory of Cancer Prevention and Therapy, Tianjin, China; 3 Tianjin’s Clinical Research Center for Cancer, Tianjin, China; 4 Department of Nursing, Tongji Hospital, Tongji Medical College, Huazhong University of Science and Technology, Wuhan, China; 5 Department of Integrated Traditional Chinese and Western Medicine, Tianjin Medical University Cancer Institute and Hospital, National Clinical Research Center for Cancer, Tianjin, China

**Keywords:** botanical drug, EGFR inhibitors, honeysuckle therapy, meta-analysis, rash, systematic review, traditional Chinese medicine

## Abstract

**Background:**

Epidermal growth factor receptor inhibitors (EGFRIs) -related rash is prevalent, and honeysuckle therapy (HT) has been extensively utilized for skin rash. However, high-quality clinical evidence supporting the use of HT for EGFRIs-related rash remains scarce. This study aims to systematically assess the prophylactic and therapeutic effectiveness and safety of HT in managing EGFRIs-related rash.

**Methods:**

A systematic literature search was performed in six electronic databases from their inception to 25 March 2025 to identify eligible randomized controlled trials (RCTs). Methodological risk of bias was evaluated using the Cochrane RoB 1.0 tool, and the certainty of evidence was graded using the GRADE approach. Meta-analyses were implemented using RevMan 5.4 software.

**Results:**

Seven RCTs with a total of 534 participants were enrolled. Prophylactically, HT significantly reduced ≥ Grade 2 rash incidence (RR = 0.43, 95% CI: 0.29–0.65, *P* < 0.001; 2 RCTs) and delayed time to rash onset (MD = 8.87 days, 95% CI: 5.30–12.44 days, *P* < 0.001; 1 RCT). No significant differences were detected in overall rash incidence (RR = 0.84, 95% CI: 0.69–1.01, *P* = 0.07; 2 RCTs) or rash duration (MD = −4.43 days, 95% CI: −9.69 to 0.83 days, *P* = 0.10; 1 RCT). For therapeutic use, HT significantly improved the clinical effective rate of rash management (RR = 1.35, 95% CI: 1.13–1.62, *P* = 0.001; 5 RCTs). Subgroup analyses confirmed consistent efficacy across single-botanical drug/compound formulations and topical/combined oral–topical administration routes. HT also improved quality of life. No severe HT-related adverse events were documented among studies that reported safety outcomes. Evidence certainty was low to very low.

**Conclusion:**

Due to the low certainty and very low certainty of evidence, HT may alleviate severe EGFRIs-related rash and improve therapeutic response. High-quality, large-scale, multicenter RCTs with standardized honeysuckle botanical drug formulations, dosages, and outcome measures are urgently needed to validate these preliminary findings and improve clinical generalizability.

**Systematic Review Registration:**

https://www.crd.york.ac.uk/PROSPERO/, Identifier CRD420251035535.

## 1 Introduction

Epidermal growth factor receptor (EGFR) activation and overexpression act as key driving factors in the occurrence and development of various malignant tumors, making EGFR a core molecular target for contemporary anticancer treatment (Ayati et al., 2020). Epidermal growth factor receptor inhibitors (EGFRIs) consist of monoclonal antibodies (targeting the extracellular domain) and small-molecule tyrosine kinase inhibitors (binding to the intracellular catalytic region), both of which have reshaped the landscape of targeted cancer therapy (Sabbah et al., 2020; Sharma et al., 2021). Currently, EGFRIs are recommended as first-line therapy for multiple malignancies with actionable molecular biomarkers, including non-small cell lung cancer (NSCLC) and colorectal cancer (González et al., 2024; Kim et al., 2025).

Despite favorable antitumor efficacy and reduced systemic toxicity relative to conventional chemotherapy, EGFRIs are associated with highly prevalent cutaneous toxicities, including acneiform rash, nail and hair changes, xerosis, and pruritus (Ibraheim et al., 2022; Yuan and Wang, 2023). Cutaneous rash is the most frequent adverse event, with an incidence as high as 92.4% (Urata et al., 2016), predominantly affecting sebaceous gland–rich areas (face, scalp, chest) and causing pain, pruritus, and psychological distress (Braden and Anadkat, 2016). Beyond physical and psychological distress, severe rash increases the risk of secondary infection and often necessitates dose reduction, interruption, or permanent discontinuation of EGFRIs, directly compromising long-term oncologic outcomes (Hayashi et al., 2019). Proactive prophylaxis and timely management of EGFRIs-related rash are therefore clinically critical to maintain treatment adherence and efficacy.

Current standard interventions for EGFRIs-induced cutaneous toxicity, including topical corticosteroids and oral tetracycline antibiotics, provide limited efficacy and are restricted by treatment-related adverse reactions (Ashida et al., 2023; You et al., 2024). Emerging therapeutic approaches such as topical Janus kinase inhibition represent ongoing advances but remain under investigation (You et al., 2024). Honeysuckle (*Lonicera japonica*), a classic traditional Chinese medicine (TCM) botanical drug recorded in ancient pharmacopoeias including Shennong’s Classic of Materia Medica and Compendium of Materia Medica, has been widely applied for thousands of years in the treatment of inflammatory and infectious skin diseases. Its ethnopharmacological functions focus on “qing re jie du” (clearing heat and detoxification) and “liao chuang zhi yang” (treating sores and relieving itching) (Liu et al., 2022). Pharmacological studies have confirmed that honeysuckle exhibits multiple beneficial activities including antioxidant, anti-inflammatory, antimicrobial, and antiviral effects, which are mediated by metabolites such as chlorogenic acid, luteolin, and quercetin (Chen et al., 2012; Cao et al., 2023).

Despite its long clinical history, rigorous systematic review and meta-analytic evidence for honeysuckle therapy (HT) in EGFRIs-related rash remains scarce. This study therefore comprehensively evaluates the prophylactic and therapeutic effects of HT aiming to provide robust ethnopharmacological insights and evidence-based supportive care for this common and impactful targeted therapy-related adverse event.

## 2 Methods

This systematic review was registered prospectively in PROSPERO with registration number CRD420251035535 and was reported in compliance with the 2020 PRISMA guidelines (Page et al., 2021).

### 2.1 Search strategy

A systematic literature search was conducted in PubMed, Embase, The Cochrane Library, CNKI, CBM, and Wanfang databases from inception to 25 March 2025. Manual screening of reference lists from included studies and related systematic reviews was performed to minimize publication bias. All retrieved records were restricted to human clinical trials, with no limitations imposed on publication language. The full search strings applied for each database are detailed in Supplementary Table S1.

### 2.2 Inclusion criteria

#### 2.2.1 Types of participants

Adult cancer patients (≥18 years) receiving any class of EGFRIs (monoclonal antibodies/tyrosine kinase inhibitors), regardless of tumor site or disease stage. Participants under 18 years of age were excluded because EGFRIs are predominantly approved and prescribed for adult cancer populations, with very limited pediatric indications; furthermore, skin toxicity profiles and clinical management differ substantially between pediatric and adult populations, which would introduce significant clinical heterogeneity.

#### 2.2.2 Types of interventions

The experimental group received honeysuckle-based interventions, including single botanical drug or compound preparations. All eligible honeysuckle regimens were aqueous decoctions of *Lonicerae Japonicae Flos* (*L. japonica* Thunb.) meeting the standards specified in the Chinese Pharmacopoeia. Per ConPhyMP guidelines (Heinrich et al., 2022), these preparations are classified as Type A extracts with consistent botanical origin and characteristic core bioactive metabolites, including chlorogenic acid, luteolin, cynaroside and quercetin. Decoctions were prepared by mixing dried floral buds with purified water, heated to boil and then simmered gently for extraction; administration routes included oral intake, topical wet compress, or combined application at variable doses, frequencies and treatment durations. The control group received routine clinical treatment, routine care, or placebo without honeysuckle.

#### 2.2.3 Types of outcomes

Primary outcomes included the incidence of EGFRIs-induced rash (graded by CTCAE) and the clinical effective rate of rash treatment. Secondary outcomes included time to rash onset, rash duration, health-related quality of life (HRQoL), and treatment-related adverse events.

#### 2.2.4 Types of studies

We only included randomized controlled trials (RCTs) in this systematic review.

### 2.3 Exclusion criteria

Studies were excluded if full text was unavailable, outcome data were incomplete, or HT was combined with unreported or untested herbal agents.

### 2.4 Study selection and data extraction

Records retrieved from all databases were imported into NoteExpress 4.1.0 software. Duplicate records were first removed, and the remaining studies were independently screened for eligibility by two authors (Lifeng Liu and Shuang Liu) based on the predefined inclusion and exclusion criteria. Discrepancies were resolved via consensus discussion or consultation with a third senior reviewer (Fangyuan Zhang).

Two reviewers (LL and SL) independently extracted data from all included RCTs using a pre-designed and pilot-tested standardized form. Extracted items included study characteristics, patient demographics, type of EGFRIs, intervention details, control measures, outcome data, and adverse events. Missing data were requested from corresponding authors via email; no responses were received, so only available data were analyzed. Any disagreements were settled by discussion or third-party adjudication.

### 2.5 Risk of bias assessment

Two authors independently assessed the methodological quality of included studies using the Cochrane RoB 1.0 tool. This tool evaluates six domains: random sequence generation, allocation concealment, blinding of participants and personnel, blinding of outcome assessment, incomplete outcome data, and selective reporting (Higgins et al., 2011). Each domain was graded as high, unclear, or low risk. Discrepancies were resolved through group discussion. Of note, although rash grading follows standardized CTCAE criteria, outcome assessment in unblinded studies may still be subject to observer bias; therefore, detection bias was rated as unclear risk for studies without explicit blinding of outcome assessors.

### 2.6 Statistical analysis

Statistical analyses were performed using RevMan 5.4 software. For dichotomous outcomes, pooled data were presented as relative risk (RR) with 95% confidence intervals (CI). For continuous outcomes, mean differences (MDs) or standardized mean differences (SMDs) with 95% CIs were used. Heterogeneity was tested by the *χ* ^*2*^ tests and *I* ^*2*^ statistics; significant heterogeneity defined as *P* < 0.10 or *I* ^*2*^ > 50%. A fixed-effect model was used for low heterogeneity, while a random-effect model was applied for substantial heterogeneity. Predefined subgroup analyses were conducted to explore sources of heterogeneity. Sensitivity analysis was performed by omitting one study at a time. Publication bias was only assessed for outcomes including ≥10 studies. All tests were two-sided (α = 0.05).

### 2.7 Certainty of evidence

The certainty of evidence was evaluated by the GRADE framework (Balshem et al., 2011). Two independent reviewers systematically graded methodological quality and classified evidence into four levels: high, moderate, low, or very low. RCTs were initially categorized as high-certainty evidence and then downgraded based on five predefined factors: risk of bias, inconsistency, imprecision, indirectness, and publication bias.

## 3 Results

### 3.1 Study selection

**FIGURE 1:**
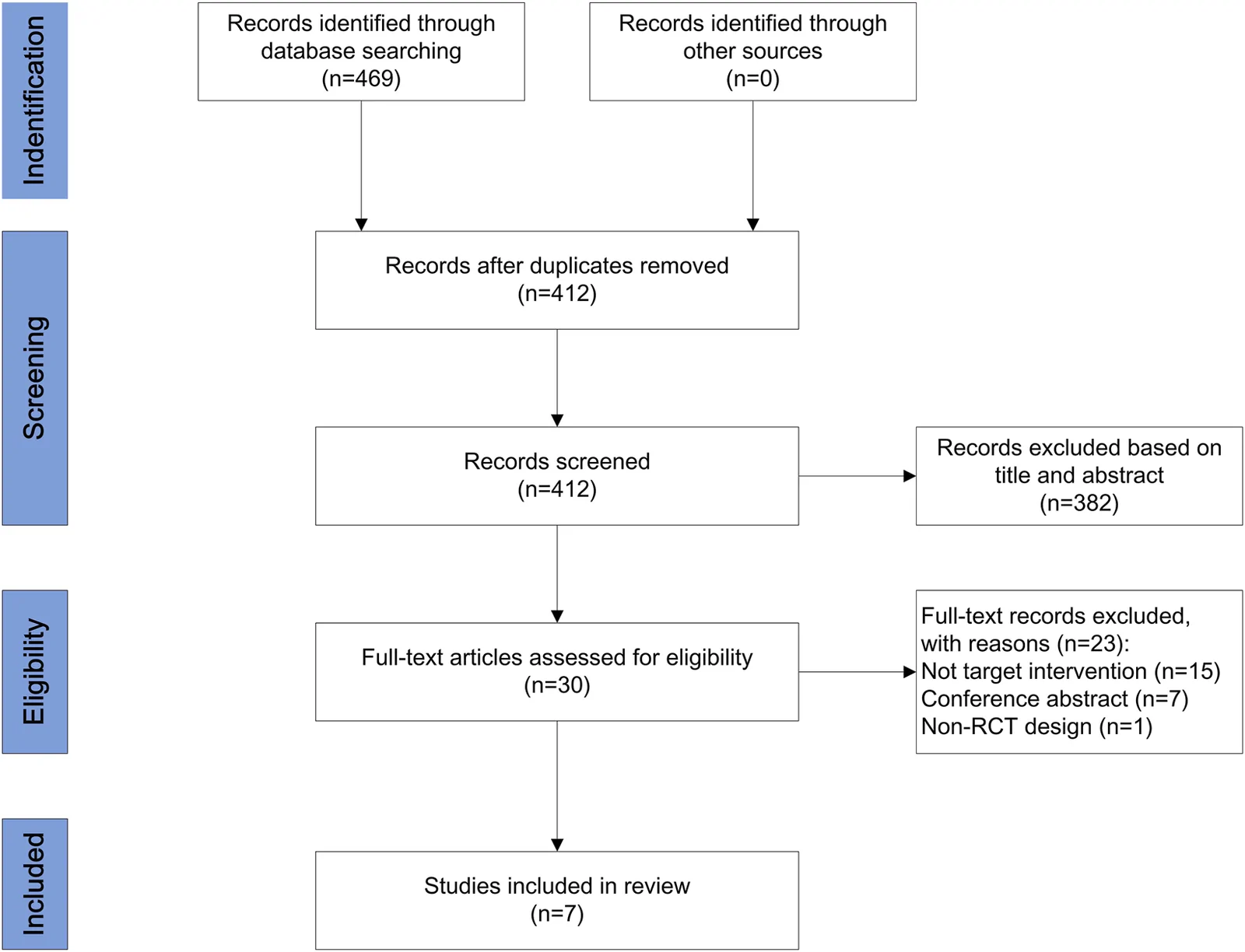
PRISMA flow diagram of study selection.

A total of 469 records were initially identified through database searching. After removing duplicates, 412 unique studies remained for screening. Among these, 382 studies were excluded based on title and abstract screening, and 30 studies underwent full-text evaluation. Finally, 23 studies were excluded for various reasons, leaving seven eligible RCTs for inclusion in this meta-analysis (Zhang et al., 2010; Lin and Deng, 2012; Jiang and Yang, 2015; Wu et al., 2017; Li et al., 2017; Lu et al., 2020; Liu et al., 2022). The PRISMA flow diagram of study selection is displayed in Figure 1.

### 3.2 Study characteristics

Seven RCTs (534 patients) were included, all conducted in China. Two studies focused on prophylaxis (Lin and Deng, 2012; Liu et al., 2022), six on therapy (Zhang et al., 2010; Jiang and Yang, 2015; Wu et al., 2017; Li et al., 2017; Lu et al., 2020; Liu et al., 2022), and one study evaluated both endpoints. Included patients had NSCLC, colorectal cancer, or head and neck cancer, receiving EGFRIs (cetuximab, erlotinib, gefitinib, afatinib). Compound formulations combined HT with Sophora flavescens Aiton., a TCM botanical drug with established anti-inflammatory and antipruritic effects for inflammatory skin diseases. Baseline and intervention characteristics are summarized in Table 1.

**TABLE 1:** Characteristics information of included studies.

Author (year)	Country	Study population	Type of EGFRIs	Sample size (E/C)	Intervention group	Control group	Intervention regimen	Main outcomes	Adverse events
Liu et al. (2022)	China	Metastatic colorectal and NSCLC	Cetuximab, erlotinib, gefitinib, icotinib	PG: 46/46 TG: 47/46	Decocted honeysuckle (prophylactic + therapeutic)	Routine treatment	Oral: 10 g honeysuckle decocted in 200 mL water, twice daily Topical: 50 g honeysuckle decocted in 1 L water, wet compress for 15 min per session, three times daily	Rash incidence (CTCAE)	Grade 1 gastrointestinal discomfort (n = 3)
Lu et al. (2020)	China	Lung cancer	Erlotinib, gefitinib or afatinib	42/42	Compound honeysuckle decoction + routine treatment	Routine treatment	Topical: 30 g honeysuckle combined with 30 g Sophora flavescens decocted in 3 L water, topical wet compress twice daily for 4 consecutive weeks	Clinical effective rate, GQOL-74	Not reported
Li et al. (2017)	China	Lung cancer	Erlotinib	19/17	Decocted honeysuckle	Routine treatment	Oral: 10 g honeysuckle decoction daily; topical: 20 g honeysuckle decoction wet compress once daily for 7 days	Clinical effective rate, KPS	Not reported
Wu et al. (2017)	China	Lung cancer	Gefitinib	40/40	Compound honeysuckle decoction + routine treatment	Routine treatment	Topical: 30 g honeysuckle combined with 30 g Sophora flavescens decocted in 3 L water, topical wet compress for 20 min per session, 2–3 times daily for 30 consecutive days	Clinical effective rate, PDI	No adverse events
Jiang and Yang (2015)	China	Colorectal, head and neck cancer	Cetuximab	31/30	Decocted honeysuckle + routine treatment	Warm water compress + routine treatment	Topical: 100 g honeysuckle decocted in 2 L water, topical wet compress for 20–30 min per session, 3–6 times daily	Clinical effective rate	No adverse events
Lin and Deng (2012)	China	Colorectal, lung cancer, etc.	Cetuximab	60/32	Decocted honeysuckle (prophylaxis)	Routine prophylaxis	Topical: 50 g honeysuckle decocted in 1 L water, topical wet compress twice daily	Rash incidence, onset time, duration	Not reported
Zhang et al. (2010)	China	Head and neck cancer	Cetuximab	23/19	Decocted honeysuckle + routine treatment	Warm water compress + routine treatment	Topical: 50 g honeysuckle decocted in 1 L water, topical wet compress for 10–20 min per session, 2–5 times daily	Clinical effective rate	Not reported

Abbreviations: C, control group; CTCAE: common terminology criteria for adverse events; E, experimental group; EGFRIs: Epidermal growth factor receptor inhibitors; GQOL-74: Generic quality of life inventory 74; KPS: karnofsky performance status; NSCLC: non-small cell lung cancer; PDI: psoriasis disability index; PG: prophylactic group; TG: treatment group.

### 3.3 Risk of bias assessment

**FIGURE 2:**
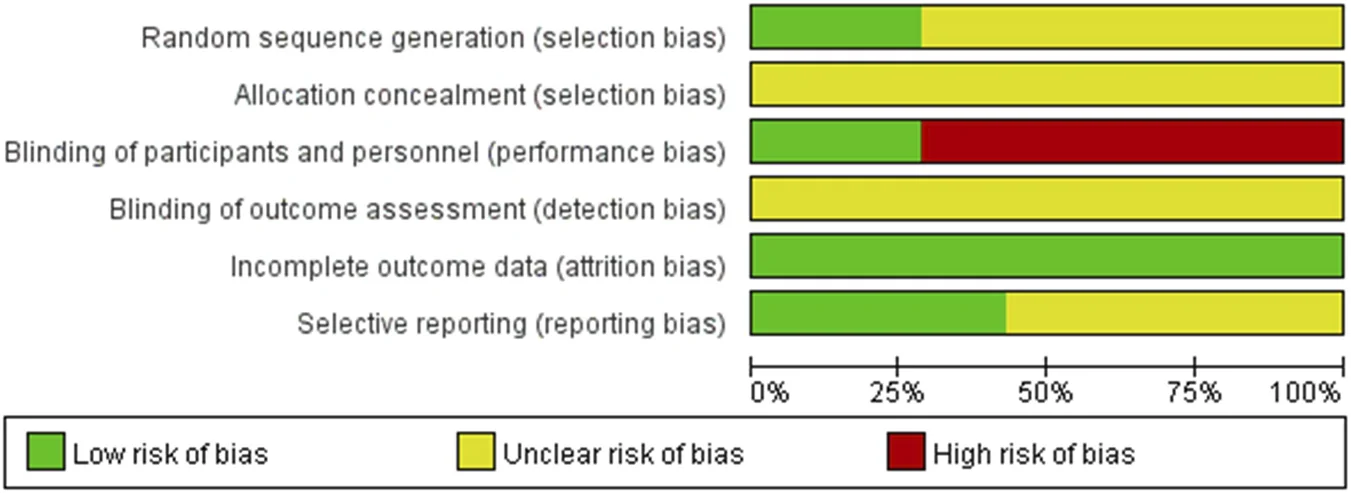
Risk of bias graph.

For random sequence generation, based on seven studies reviewed, we found that Zhang et al. (2010) used a random number table, Wu et al. (2017) employed a drawing method, and other studies did not provide details on their randomization approaches. None of the included RCTs described allocation concealment. Only two RCTs (Zhang et al., 2010; Jiang and Yang, 2015) adopted a placebo-controlled design, while the other studies were open-label and therefore carried unclear or high risk of performance bias. Detection bias was rated as unclear risk for all studies, as explicit blinding of outcome assessors was not consistently reported; although CTCAE provides objective grading criteria, clinical observer judgment may still introduce bias in unblinded settings. No patient attrition was reported in any study. Studies that did not report adverse events were rated as unclear risk of selective reporting (Zhang et al., 2010; Lin and Deng, 2012; Li et al., 2017; Lu et al., 2020); others were rated as low risk. The overall risk of bias is presented in Figure 2.

### 3.4 Primary outcomes

#### 3.4.1 Incidence of EGFRIs-related rash

**FIGURE 3:**
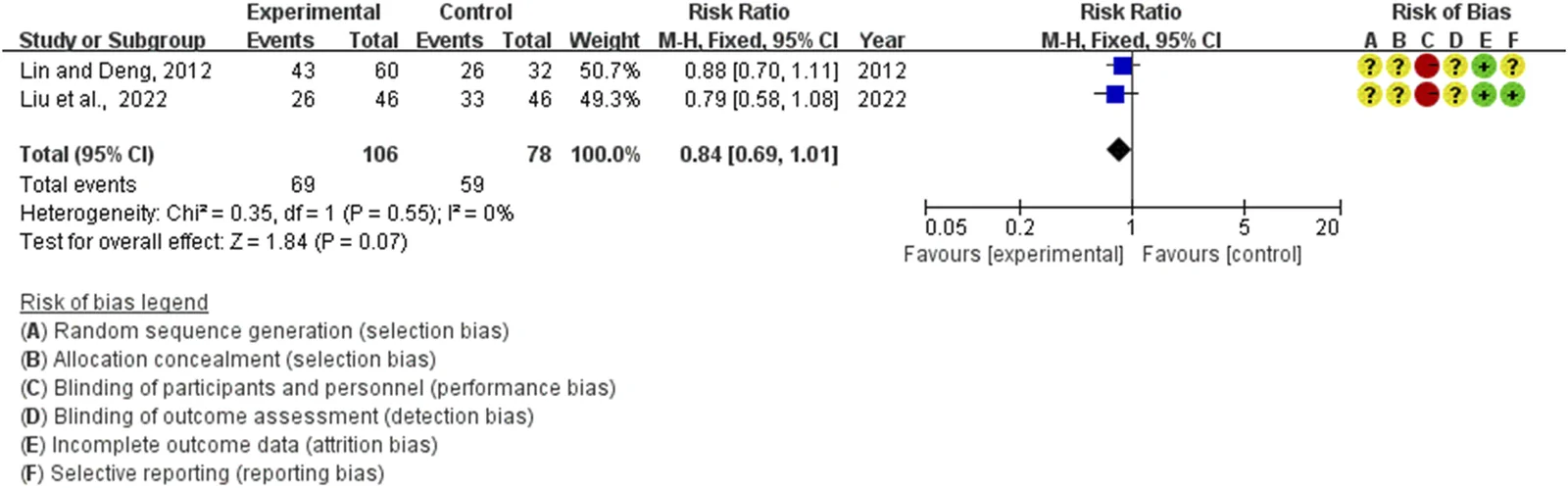
Forest plot of HT on overall incidence of EGFRIs-related rash.

**FIGURE 4:**
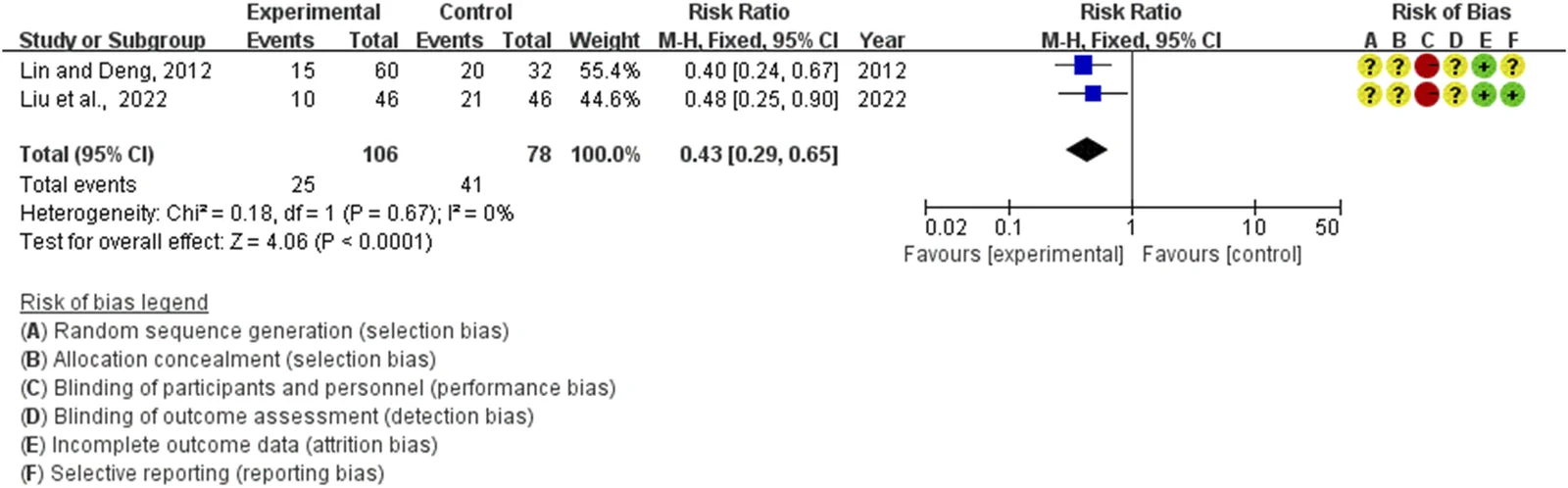
Forest plot of HT on incidence of ≥Grade 2 EGFRIs-related rash.

Two studies (Lin and Deng, 2012; Liu et al., 2022) reported overall rash incidence (184 patients). Meta-analysis showed no significant difference between HT and control groups (RR = 0.84, 95% CI: 0.69–1.01, *P* = 0.07), with no heterogeneity (*I* ^*2*^ = 0%). Two studies (Lin and Deng, 2012; Liu et al., 2022) reported ≥Grade 2 rash incidence (184 patients). Pooled results indicated that HT significantly reduced this risk (RR = 0.43, 95% CI: 0.29–0.65, *P* < 0.001), with no heterogeneity (*P* = 0.67, *I* ^*2*^ = 0%). Forest plots are shown in Figures 3 and 4.

#### 3.4.2 Clinical efficacy rate of rash treatment

**FIGURE 5:**
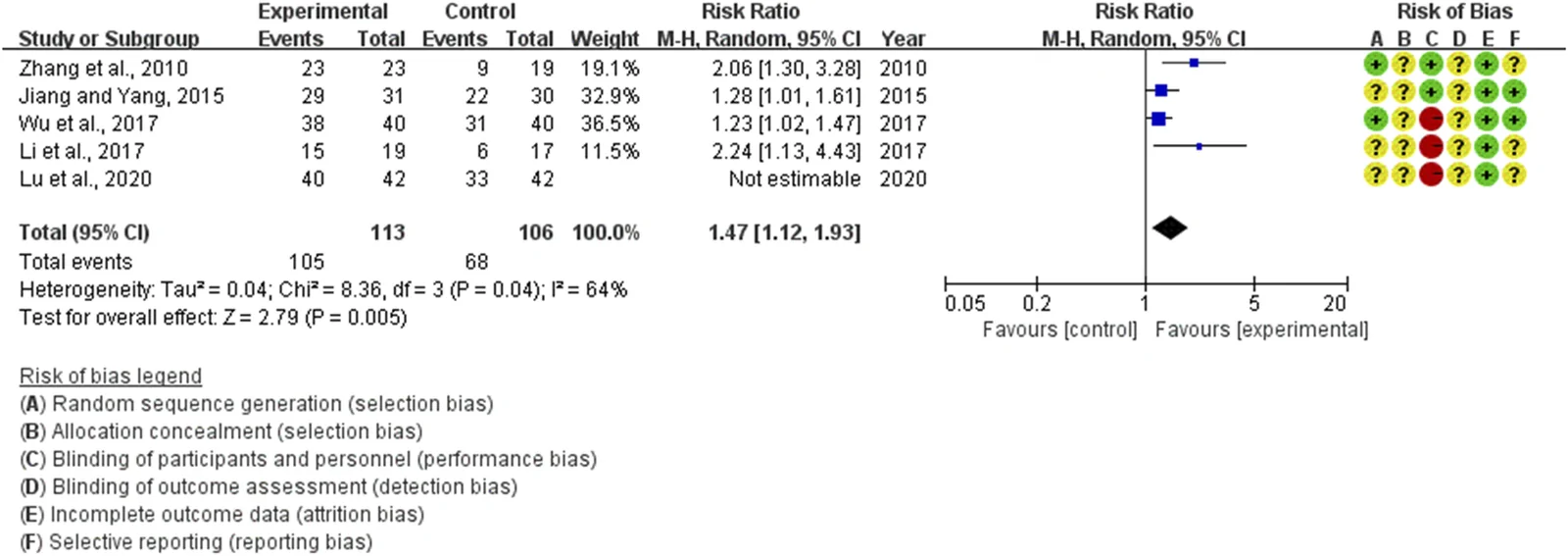
Forest plot of clinical effective rate of HT for EGFRIs-related rash.

Five studies with 303 patients were included in this analysis (Zhang et al., 2010; Jiang and Yang, 2015; Wu et al., 2017; Li et al., 2017; Lu et al., 2020). HT significantly improved the clinical effective rate compared with control (RR = 1.35, 95% CI = 1.13–1.62, *P* = 0.001). Moderate heterogeneity was observed (*I* ^*2*^ = 55%, *P* = 0.06), so a random-effects model was used. Sources of heterogeneity may include differences in EGFRI type, honeysuckle preparation formulation, dosage, and administration route (oral vs. topical vs. combined). Sensitivity analysis confirmed the robustness of the result (Supplementary Table S2). Forest plot is displayed in Figure 5.

**FIGURE 6:**
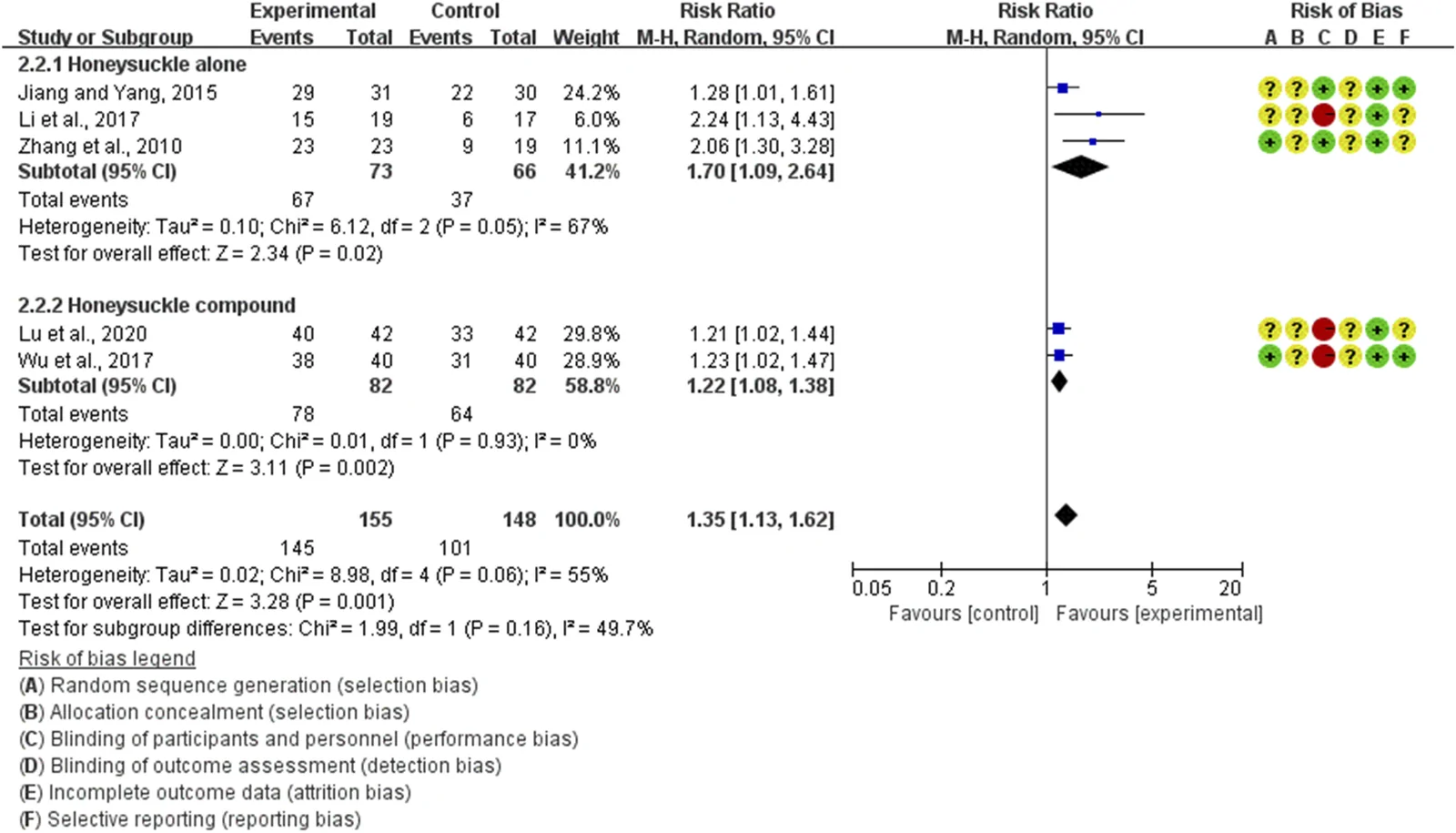
Subgroup analysis of single botanical drug vs compound HT for clinical effective rate.

**FIGURE 7:**
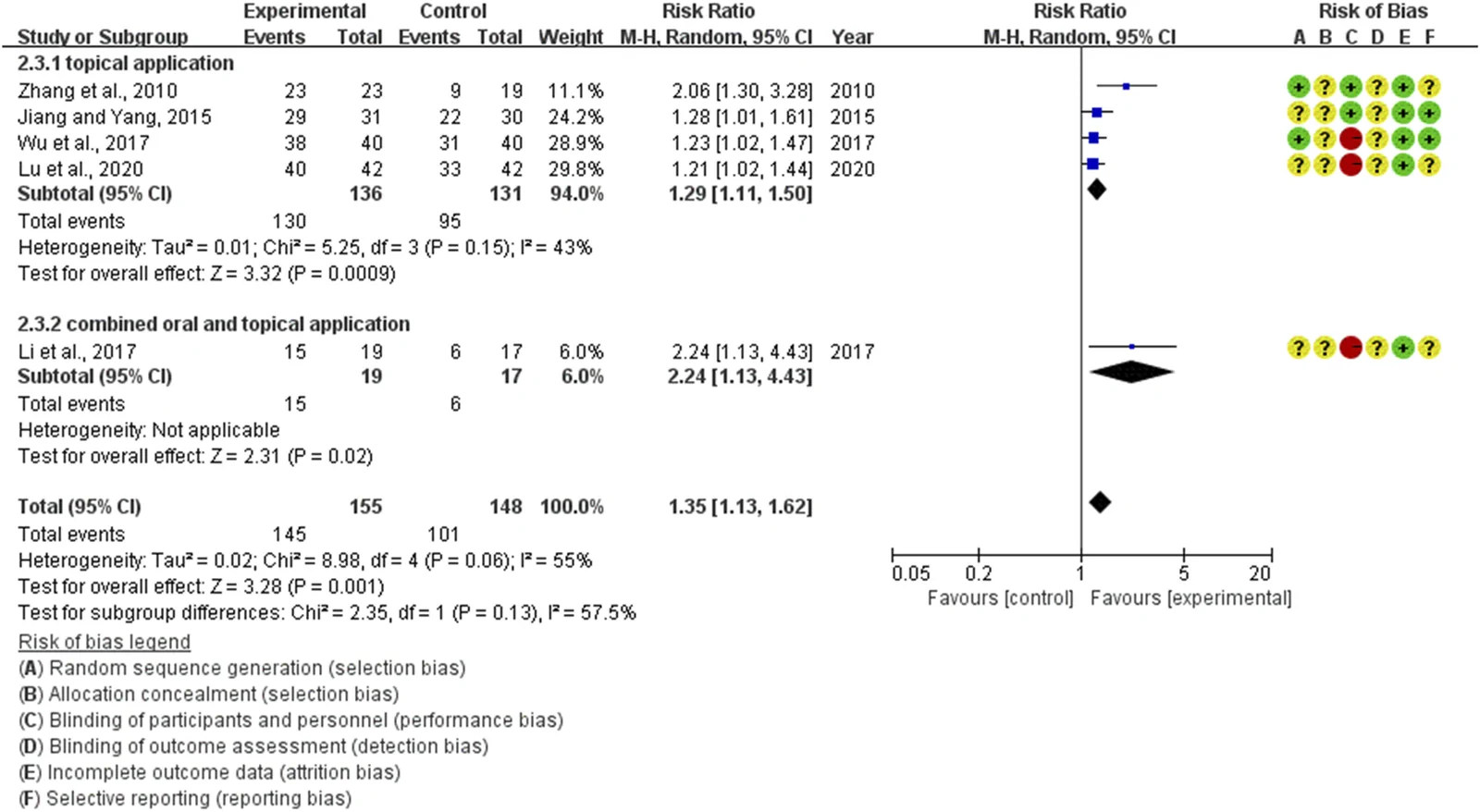
Subgroup analysis of topical vs combined oral–topical HT for clinical effective rate.

Subgroup analyses were performed based on formulation (single botanical drug vs. compound) and administration route (topical vs. combined oral–topical). Results showed consistent beneficial effects across all subgroups, indicating stable efficacy regardless of preparation form or delivery route. Forest plots of subgroup analyses are shown in Figures 6 and 7.

### 3.5 Secondary outcomes

#### 3.5.1 Time to rash onset and rash duration

Data on the onset and duration of EGFRIs-related rash were only reported in one study with 92 patients (Lin and Deng, 2012). Thus, no meta-analytic pooling was performed for these outcomes. Within this single trial, the HT group exhibited a delayed rash onset by 8.87 days compared with controls (95% CI: 5.30–12.44 days, *P* < 0.001). No significant difference in rash duration was found between groups (MD = −4.43 days, 95% CI: −9.69 to 0.83 days, *P* = 0.10).

#### 3.5.2 HRQoL

Three studies (Wu et al., 2017; Li et al., 2017; Lu et al., 2020) assessed HRQoL using different validated scales, with one of which employed reverse scoring. Thus, a meta-analysis was not conducted. Despite the variation in measurement tools, all studies consistently reported statistically significant improvements in HRQoL following HT for EGFRIs-induced rash (*P* < 0.05).

#### 3.5.3 Adverse events

Only three studies reported adverse events. Among these, two studies reported no AEs (Jiang and Yang, 2015; Wu et al., 2017), only one study reported mild CTCAE Grade 1 gastrointestinal discomfort in three patients receiving oral honeysuckle decoction (Liu et al., 2022). No severe HT-related adverse events were documented among studies that reported safety outcomes. However, given that several studies did not systematically report adverse events, and that dosage, frequency, and administration routes varied considerably across included trials, the overall safety profile cannot be definitively established for any specific honeysuckle regimen. No data on drug-drug interactions between honeysuckle and EGFRIs were identified in the included studies.

### 3.6 Publication bias

Funnel plots and Egger’s test were not performed because all outcomes included fewer than 10 studies.

### 3.7 Certainty of evidence

Evidence certainty was rated from low to very low using the GRADE approach. The main reasons for downgrading were high risk of bias (insufficient blinding) and imprecision (small sample size). No downgrading was made for indirectness or publication bias. Details are listed in Table 2.

**TABLE 2:** GRADE evidence profiles.

Outcome	No. of studies	No. of patients	Risk of bias	Inconsistency	Indirectness	Imprecision	Other consideration	Effect	Certainty
Overall rash incidence	2	184	Serious^a^	Not serious	Not serious	Serious^b^	None	RR 0.84 (0.69–1.01)	⊕⊕○○ Low
≥Grade 2 rash incidence	2	184	Serious^a^	Not serious	Not serious	Serious^b^	None	RR 0.43 (0.29–0.65)	⊕⊕○○ Low
Time to rash onset	1	92	Serious^a^	Not applicable	Not serious	Very serious^b^	None	MD 8.87 days, (5.30–12.44)	⊕○○○ Very low
Rash duration	1	92	Serious^a^	Not applicable	Not serious	Very serious^b^	None	MD −4.43 days, (−9.69 to 0.83)	⊕○○○ Very low
Clinical effective rate	5	303	Serious^a^	serious^c^	Not serious	Not serious	None	RR 1.35 (1.13–1.62)	⊕⊕○○ Low
Health-related quality of life	3	200	Serious^a^	Not serious	Not serious	Very serious^b^	None	Not applicable	⊕○○○ Very low
Adverse events	3	280	Serious^a^	Not serious	Not serious	Serious^b^	None	Not applicable	⊕⊕○○ Low

^a^ Blinding of participants and personnel of most included studies rated as high.

^b^ Small sample sizes.

^c^ *I^2^* ≥ 50%.

## 4 Discussion

This systematic review and meta-analysis comprehensively evaluated the efficacy and safety of HT for EGFRIs-related rash based on seven RCTs and 534 patients. Key findings suggest that HT can reduce the incidence of ≥Grade 2 rash, delay rash onset, improve clinical response rate, and enhance quality of life, with a probably favorable safety profile based on available safety data. However, the low certainty and very low certainty of evidence may weaken the reliability of the results.

The significant reduction in ≥Grade 2 rash is clinically pivotal, as severe rash is the primary driver of EGFRIs dose reduction, interruption, or permanent discontinuation (Hayashi et al., 2019). The lack of effect on total rash incidence reflects the naturally high baseline rate of mild rash (up to 92.4%) (Urata et al., 2016), which is often self-limiting and does not require intervention. HT selectively targets clinically impactful severe cutaneous toxicity, which aligns precisely with its traditional ethnopharmacological indication for “heat-toxin-induced severe skin disorders” (Li et al., 2022; Cao et al., 2023).

Pooled analysis also suggested that HT may improve rash treatment efficacy, which is generally consistent with previous systematic reviews of Chinese botanical interventions for EGFRIs-related rash (Chen et al., 2019; Zheng et al., 2019). Subgroup analyses further validated the consistency and flexibility of HT: both single-botanical drug and compound preparations, as well as topical and combined oral–topical administration, were effective. This allows personalized clinical application based on patient tolerance, preference, and clinical setting. Although meta-analysis of HRQoL was not feasible, all included studies showed significant quality-of-life improvements, supporting the patient-centered value of HT (Hung et al., 2019).

The pharmacological mechanisms underlying the potential effects of HT are closely related to the pathological process of EGFRIs-induced rash. EGFR inhibition disrupts epidermal keratinocyte differentiation and triggers innate immune activation and release of proinflammatory cytokines, which drive acneiform rash formation. The multiple active metabolites of honeysuckle, including chlorogenic acid, luteolin, and quercetin, exert anti-inflammatory, antioxidant, and immunomodulatory effects by inhibiting proinflammatory mediators and reducing oxidative stress, which may directly alleviate the inflammatory response of skin lesions (Leung et al., 2006; Feng et al., 2021; Zheng et al., 2022). In addition, the antimicrobial activity of these metabolites helps prevent secondary bacterial infection in damaged skin, which may further support skin repair (Zhou et al., 2015; Shi et al., 2016; Cao et al., 2023).

This study has several limitations. First, the total pooled sample size of only 534 patients across seven trials is relatively small, which may weaken statistical power and restrict the broad generalizability of our findings. Second, we performed comprehensive searches across six Chinese and English electronic databases, yet no eligible RCTs investigating honeysuckle for EGFRIs-induced rash were identified outside China, reflecting that this intervention remains underexplored internationally and limiting the global generalizability of current evidence. Third, the overall methodological quality was relatively low due to unclear randomization, lack of allocation concealment, and insufficient blinding. Notably, detection bias was rated as unclear rather than low, as observer judgment in rash assessment may introduce bias in open-label studies despite standardized CTCAE criteria. Fourth, substantial clinical heterogeneity existed across trials, including different types of EGFRIs, variable honeysuckle preparations (single-botanical drug vs. compound formulation), different administration routes (oral, topical, combined), and varying dosages and treatment durations, which may reduce the comparability of pooled results. Fifth, safety data were incomplete: several studies did not systematically report adverse events, and no definitive safety conclusions can be drawn for specific dosage regimens. No data on drug-drug interactions between honeysuckle and EGFRIs were available from the included studies. Finally, long-term efficacy and mechanistic data remain lacking. Future studies should conduct large-scale, multicenter, double-blind, placebo-controlled RCTs with standardized honeysuckle botanical drug formulations, dosages, and outcome indicators. Mechanistic studies are also needed to explore the molecular pathways of HT against EGFRIs-related rash.

## 5 Conclusion

Current evidence suggests that HT may reduce the incidence of severe EGFRIs-related rash, delay time to rash onset, improve clinical therapeutic response, and enhance health-related quality of life, with a probably favorable tolerability profile based on available safety data. HT may be a potential complementary ethnopharmacological intervention for the management of EGFRIs-induced cutaneous toxicity. High-quality, large-scale, multicenter, standardized RCTs are urgently needed to confirm these findings and support broader clinical application.

## Data Availability

The original contributions presented in the study are included in the article/Supplementary Material, further inquiries can be directed to the corresponding author.

## References

[B1] Ashida A. Tomida S. Iwabuchi T. Sato Y. Kiniwa Y. Okuyama R. (2023). Persistent alteration of the skin microbiome in patients with skin rash after receiving EGFR inhibitor treatment. Exp. Dermatol. 32 (5), 671–677. 10.1111/exd.14728 36514876

[B2] Ayati A. Moghimi S. Salarinejad S. Safavi M. Pouramiri B. Foroumadi A. (2020). A review on progression of epidermal growth factor receptor (EGFR) inhibitors as an efficient approach in cancer targeted therapy. Bioorg. Chem. 99, 103811. 10.1016/j.bioorg.2020.103811 32278207

[B3] Balshem H. Helfand M. Schünemann H. J. Oxman A. D. Kunz R. Brozek J. (2011). GRADE guidelines: 3. Rating the quality of evidence. J. Clin. Epidemiol. 64 (4), 401–406. 10.1016/j.jclinepi.2010.07.015 21208779

[B4] Braden R. L. Anadkat M. J. (2016). EGFR inhibitor-induced skin reactions: differentiating acneiform rash from superimposed bacterial infections. Support. Care Cancer. 24 (9), 3943–3950. 10.1007/s00520-016-3231-1 27117557

[B5] Cao Y. X. Ji P. Wu F. L. Dong J. Q. Li C. C. Ma T. (2023). *Lonicerae Japonicae caulis*: a review of its research progress of active metabolites and pharmacological effects. Front. Pharmacol. 14, 1277283. 10.3389/fphar.2023.1277283 37954842 PMC10635453

[B6] Chen W. C. Liou S. S. Tzeng T. F. Lee S. L. Liu I. M. (2012). Wound repair and anti-inflammatory potential of *Lonicera japonica* in excision wound-induced rats. BMC Complement. Altern. Med. 12, 226. 10.1186/1472-6882-12-226 23173654 PMC3577469

[B7] Chen Z. Q. Li Z. Y. Yang C. Z. Lin R. T. Lin L. Z. Sun L. L. (2019). Chinese herbal medicine for epidermal growth factor receptor inhibitor-induced skin rash in patients with malignancy: an updated meta-analysis of 23 randomized controlled trials. Complement. Ther. Med. 47, 102167. 10.1016/j.ctim.2019.08.001 31780021

[B8] Feng J. Liu Z. Chen H. Zhang M. Ma X. Han Q. (2021). Protective effect of cynaroside on sepsis-induced multiple organ injury through Nrf2/HO-1-dependent macrophage polarization. Eur. J. Pharmacol. 911, 174522. 10.1016/j.ejphar.2021.174522 34560076

[B9] González N. S. Marchese P. V. Baraibar I. Ros J. Salvà F. Rodríguez M. (2024). Epidermal growth factor receptor antagonists in colorectal cancer: emerging strategies for precision therapy. Expert. Opin. Investig. Drugs. 33 (6), 613–625. 10.1080/13543784.2024.2349287 38775361

[B10] Hayashi H. Iihara H. Hirose C. Fukuda Y. Kitahora M. Kaito D. (2019). Effects of pharmacokinetics-related genetic polymorphisms on the side effect profile of afatinib in patients with non-small cell lung cancer. Lung Cancer 134, 1–6. 10.1016/j.lungcan.2019.05.013 31319966

[B11] Heinrich M. Jalil B. Abdel-Tawab M. Echeverria J. Kulić Ž. McGaw L. J. (2022). Best practice in the chemical characterisation of extracts used in pharmacological and toxicological research-The ConPhyMP-Guidelines. Front. Pharmacol. 13, 953205. 10.3389/fphar.2022.953205 36176427 PMC9514875

[B12] Higgins J. P. Altman D. G. Gøtzsche P. C. Jüni P. Moher D. Oxman A. D. (2011). The Cochrane Collaboration's tool for assessing risk of bias in randomised trials. BMJ 343, d5928. 10.1136/bmj.d5928 22008217 PMC3196245

[B13] Hung Y. C. Chin C. Y. Lee Y. C. Chen Y. H. Tsai M. Y. (2019). Clinical experience of Chinese herbal medicine ameliorates dermatologic events from epidermal growth factor receptor inhibitors for lung cancer: a case series. Explore (NY) 15 (5), 363–370. 10.1016/j.explore.2018.11.001 30497918

[B14] Ibraheim M. K. Lo J. Gupta R. Parseghian C. Patel A. B. (2022). Acneiform eruptions with combination targeted cancer therapy in colorectal cancer patients. Support. Care Cancer. 30 (10), 8051–8058. 10.1007/s00520-022-07257-2 35771289

[B15] Jiang X. L. Yang Z. J. (2015). Efficacy observation of Lonicera japonica solution wet dressing in the treatment of cetuximab-induced acneiform rash. China Pharm. 26 (02), 243–244.

[B16] Kim J. Park S. Ku B. M. Ahn M. J. (2025). Updates on the treatment of epidermal growth factor receptor-mutant non-small cell lung cancer. Cancer 131 (1), e35778. 10.1002/cncr.35778 40171939

[B17] Leung H. W. Kuo C. L. Yang W. H. Lin C. H. Lee H. Z. (2006). Antioxidant enzymes activity involvement in luteolin-induced human lung squamous carcinoma CH27 cell apoptosis. Eur. J. Pharmacol. 534 (1-3), 12–18. 10.1016/j.ejphar.2006.01.021 16469309

[B18] Li L. Z. Fang C. T. Zhang H. T. Liu L. W. Meng J. C. Kuang W. (2017). Clinical observation on the treatment of targeted drug-related rashes by external flower washing combined with internal administration. Hubei J. Traditional Chin. Med. 39 (2), 35–36.

[B19] Li C. L. Hsia T. C. Yang S. T. Chao K. C. Tu C. Y. Chen H. J. (2022). Efficacy of prophylactic Traditional Chinese medicine on skin toxicity of afatinib in EGFR mutation-positive advanced lung adenocarcinoma: a single-center, prospective, double-blinded, randomized-controlled pilot trial. Integr. Cancer. Ther. 21, 15347354221086663. 10.1177/15347354221086663 35297709 PMC8943309

[B20] Lin L. Deng D. H. (2012). “The decoction of honeysuckle relieves the rash caused by cetuximab,” in Proceedings of the National Symposium on New Advances in Oncology Nursing, 387–389.

[B21] Liu Z. Tian T. Wang B. Lu D. Ruan J. Shan J. (2022). Reducing acneiform rash induced by EGFR inhibitors with honeysuckle therapy: a prospective, randomized, controlled study. Front. Pharmacol. 13, 835166. 10.3389/fphar.2022.835166 35250582 PMC8894807

[B22] Lu J. Y. Chen L. J. Guo Z. Y. (2020). Observation on the effect of compound honeysuckle decoction combined with chlortetracycline eye ointment on the severity and duration of skin rash caused by EGFRIs. J. Liaoning Univ. Trad. Chin. Med. 22 (8), 191–194. 10.13194/j.issn.1673-842x.2020.08.047

[B23] Page M. J. McKenzie J. E. Bossuyt P. M. Boutron I. Hoffmann T. C. Mulrow C. D. (2021). The PRISMA 2020 statement: an updated guideline for reporting systematic reviews. Syst. Rev. 10 (1), 89. 10.1186/s13643-021-01626-4 33781348 PMC8008539

[B24] Sabbah D. A. Hajjo R. Sweidan K. (2020). Review on epidermal growth factor receptor (EGFR) structure, signaling pathways, interactions, and recent updates of EGFR inhibitors. Curr. Top. Med. Chem. 20 (10), 815–834. 10.2174/1568026620666200303123102 32124699

[B25] Sharma B. Singh V. J. Chawla P. A. (2021). Epidermal growth factor receptor inhibitors as potential anticancer agents: an update of recent progress. Bioorg. Chem. 116, 105393. 10.1016/j.bioorg.2021.105393 34628226

[B26] Shi Z. Liu Z. Liu C. Wu M. Su H. Ma X. (2016). Spectrum-effect relationships between chemical fingerprints and antibacterial effects of Lonicerae Japonicae flos and Lonicerae flos base on UPLC and microcalorimetry. Front. Pharmacol. 7, 12. 10.3389/fphar.2016.00012 26869929 PMC4735347

[B27] Urata Y. Katakami N. Morita S. Kaji R. Yoshioka H. Seto T. (2016). Randomized phase III study comparing gefitinib with erlotinib in patients with previously treated advanced lung adenocarcinoma: WJOG 5108L. J. Clin. Oncol. 34 (27), 3248–3257. 10.1200/JCO.2015.63.4154 27022112

[B28] Wu H. L. Li Y. Ning X. Y. Ma H. J. Zhang M. M. Tian T. (2017). Clinical efficacy of compound honeysuckle decoction hot and humid joint fusidic acid cream in treatment of targeted drugs-induced rash. Prog. Mod. Biomed. 17 (27), 5258–5261. 10.13241/j.cnki.pmb.2017.27.014

[B29] You Q. Chen L. Li S. Liu M. Tian M. Cheng Y. (2024). Topical JAK inhibition ameliorates EGFR inhibitor-induced rash in rodents and humans. Sci. Transl. Med. 16 (752), eabq7074. 10.1126/scitranslmed.abq7074 38896602

[B30] Yuan C. Wang B. (2023). Acneiform eruption induced by molecularly targeted agents in antineoplastic therapy: a review. J. Cosmet. Dermatol. 22 (8), 2150–2157. 10.1111/jocd.15704 36924348

[B31] Zhang H. L. Wang G. R. Zhang S. B. Xiao H. Y. Zhong Y. X. Lang J. Y. (2010). The efficacy of wet compress with flos lonicerae for cetuximab correlative erythra. Chin. J. Nurs. 45 (4), 307–310. 10.3761/j.issn.0254-1769.2010.04.007

[B32] Zheng S. Y. Cui H. J. Peng Y. M. Li Q. Shen W. Zhang J. Y. (2019). Efficacy of Traditional Chinese medicine in the treatment of rash caused by epidermal growth factor receptor inhibitors: a frequency statistics and meta-analysis. World J. Trad. Chin. Med. 5 (4), 269–275. 10.4103/wjtcm.wjtcm_32_19

[B33] Zheng S. Liu S. Hou A. Wang S. Na Y. Hu J. (2022). Systematic review of Lonicerae Japonicae flos: a significant food and traditional Chinese medicine. Front. Pharmacol. 13, 1013992. 10.3389/fphar.2022.1013992 36339557 PMC9626961

[B34] Zhou Z. Li X. Liu J. Dong L. Chen Q. Liu J. (2015). Honeysuckle-encoded atypical microRNA2911 directly targets influenza A viruses. Cell Res. 25 (1), 39–49. 10.1038/cr.2014.130 25287280 PMC4650580

