# Supplementary material for: Safety and efficacy of honeysuckle therapy for epidermal growth factor receptor inhibitor-related rash: a systematic review and meta-analysis
Source: Front Pharmacol. 2026 Aug 31;17:1875649. doi: 10.3389/fphar.2026.1875649 (PMC13572728; doi:10.3389/fphar.2026.1875649)
Supplement: Supplementary file 2 [file Table1.docx]

**Search strategy of each database**

1. **PubMed**

Search data: 25/3/2025

| 1 | "Exanthema"[MeSH Terms] | 10093 |
| --- | --- | --- |
| 2 | "Lonicera"[MeSH Terms] | 910 |
| 3 | “honeysuckle”[Title/Abstract] OR “honeysuckles”[Title/Abstract]) OR “loniceras”[Title/Abstract] OR “lonicera”[Title/Abstract] | 1446 |
| 4 | "exanthema"[Title/Abstract] OR "exanthem"[Title/Abstract] OR "rash"[Title/Abstract] OR "skin rash"[Title/Abstract] | 38740 |
| 5 | #1 OR #4 | 42871 |
| 6 | #2 OR #3 | 1650 |
| 7 | #5 AND #6 | 4 |

2. **Embase**

Search data: 25/3/2025

| 1 | 'rash'/exp | 170120 |
| --- | --- | --- |
| 2 | 'lonicera'/exp | 1263 |
| 3 | rash:ti,ab,kw OR exanthem:ti,ab,kw OR exanthema:ti,ab,kw OR 'skin rash':ti,ab,kw OR 'skin eruption':ti,ab,kw | 70487 |
| 4 | lonicera:ti,ab,kw OR loniceras:ti,ab,kw OR honeysuckle:ti,ab,kw OR honeysuckles:ti,ab,kw | 1130 |
| 5 | #1 OR #3 | 186568 |
| 6 | #2 OR #4 | 1555 |
| 7 | #5 AND #6 | 12 |

3. **Cochrane**

Search data: 25/3/2025

| 1 | MeSH descriptor: [Exanthema] explode all trees | 297 |
| --- | --- | --- |
| 2 | MeSH descriptor: [Lonicera] explode all trees | 6 |
| 3 | (honeysuckle):ti,ab,kw OR (honeysuckles):ti,ab,kw OR (lonicera):ti,ab,kw OR (loniceras):ti,ab,kw | 70 |
| 4 | (exanthema):ti,ab,kw OR (exanthem):ti,ab,kw OR ("skin rash"):ti,ab,kw OR (rash):ti,ab,kw | 10754 |
| 5 | #1 OR #4 | 10754 |
| 6 | #2 OR #3 | 70 |
| 7 | #5 AND #6 | 6 |

4. **CBM**

Search data: 25/3/2025

| 1 | "疹"[不加权:扩展] | 8469 |
| --- | --- | --- |
| 2 | "金银花"[不加权:扩展] | 1713 |
| 3 | "金银花"[常用字段:智能] OR "双花"[常用字段:智能] OR "银花"[常用字段:智能] | 8313 |
| 4 | "皮疹"[常用字段:智能] OR "疹子"[常用字段] OR "疹"[常用字段:智能] | 156636 |
| 5 | #4 OR #1 | 156636 |
| 6 | #3 OR #2 | 8313 |
| 7 | #6 AND #5 | 384 |
| 8 | (#6 AND #5) AND ( 临床试验[文献类型] OR 随机对照试验[文献类型]) | 109 |

5. **Wanfang**

Search data: 25/3/2025

| 1 | (题名或关键词:(皮疹) or 题名或关键词:(疹子) or 题名或关键词:(疹)) and (题名或关键词:(金银花) or 题名或关键词:(双花) or 题名或关键词:(银花) or 题名或关键词:(忍冬花)) | 155 |
| --- | --- | --- |

6. **CNKI**

Search data: 25/3/2025

| 1 | 皮疹[主题] OR 疹子[主题] OR 疹[主题] | 49508 |
| --- | --- | --- |
| 2 | 金银花[主题] OR 忍冬花[主题] OR 双花[主题] OR 银花[主题] | 20115 |
| 3 | #1 AND #2 | 183 |
