# Supplementary material for: Safety and efficacy of honeysuckle therapy for epidermal growth factor receptor inhibitor-related rash: a systematic review and meta-analysis
Source: Front Pharmacol. 2026 Aug 31;17:1875649. doi: 10.3389/fphar.2026.1875649 (PMC13572728; doi:10.3389/fphar.2026.1875649)
Supplement: Supplementary file 3 [file Table2.docx]

**Sensitivity analysis**

| **Outcome** | **Omitted Study** | **Data with study removed RR/SMD (95% CI)** | ***P*** | ***I^2^*** |
| --- | --- | --- | --- | --- |
| clinical effective rate of rash treatment | None | 1.35(1.13, 1.62) | 0.001 | 55% |
|  | Zhang et al., 2010 | 1.26(1.11, 1.42) | <0.001 | 15% |
|  | Jiang and Yang, 2015 | 1.43 (1.10, 1.85) | 0.007 | 68% |
|  | Wu et al., 2017 | 1.47 (1.11, 1.93) | 0.006 | 66% |
|  | Li et al., 2017 | 1.29 (1.11, 1.50) | <0.001 | 43% |
|  | Lu et al., 2020 | 1.47(1.12, 1.93) | 0.005 | 64% |
